# A Maize Jasmonate Zim-Domain Protein, ZmJAZ14, Associates with the JA, ABA, and GA Signaling Pathways in Transgenic *Arabidopsis*

**DOI:** 10.1371/journal.pone.0121824

**Published:** 2015-03-25

**Authors:** Xiaojin Zhou, Shengwei Yan, Cheng Sun, Suzhen Li, Jie Li, Miaoyun Xu, Xiaoqing Liu, Shaojun Zhang, Qianqian Zhao, Ye Li, Yunliu Fan, Rumei Chen, Lei Wang

**Affiliations:** 1 Department of Crop Genomics & Genetic Improvement, Biotechnology Research Institute, Chinese Academy of Agricultural Sciences, Beijing, China; 2 National Key Facility for Crop Gene Resources and Genetic Improvement, Beijing, China; 3 Department of Agronomy, Agricultural University of Hebei/Hebei Sub-center of Chinese National Maize Improvement Center, Baoding, China; 4 College of Life Sciences, Beijing Normal University, Beijing, China; University of Delhi South Campus, INDIA

## Abstract

Jasmonate (JA) is an important signaling molecule involved in the regulation of many physiological and stress-related processes in plants. Jasmonate ZIM-domain (JAZ) proteins have been implicated in regulating JA signaling pathways and the cross talk between various phytohormones. Maize is not only an important cereal crop, but also a model plant for monocotyledon studies. Although many JAZ proteins have been characterized in *Arabidopsis* and rice, few reports have examined the function of JAZ proteins in maize. In this report, we examined the phylogenetic relationship and expression pattern of *JAZ* family genes in maize. In addition, a tassel and endosperm-specific *JAZ* gene, *ZmJAZ14*, was identified using microarray data analysis and real-time RT-PCR, and its expression was induced by polyethylene glycol (PEG), jasmonate (JA), abscisic acid (ABA), and gibberellins (GAs). ZmJAZ14 was shown to be localized in the nucleus and possessed no transcriptional activating activity, suggesting that it functions as a transcriptional regulator. We found that overexpression of *ZmJAZ14* in *Arabidopsis* enhanced plant tolerance to JA and ABA treatment, as well as PEG stress, while it promoted growth under GA stimulus. Moreover, ZmJAZ14 interacted with a subset of transcription factors in *Arabidopsis*, and the accumulation of several marker genes involved in JA, ABA, and GA signaling pathways were altered in the overexpression lines. These results suggest that ZmJAZ14 may serve as a hub for the cross talk among the JA, ABA, and GA signaling pathways. Our results can be used to further characterize the function of JAZ family proteins in maize, and the gene cloned in this study may serve as a candidate for drought tolerance and growth promotion regulation in maize.

## Introduction

Plant hormones are implicated in many aspects of plant growth and development, as well as in the response to environmental cues. Both positive and negative regulators are involved in hormonal signal-transduction pathways to mediate various pathways in response to the same stimulus. Therefore, cross talk between various signaling pathways is essential to balance developmental and stress responses. Jasmonates (JAs), a class of fatty acid-derived hormone molecules, are important signaling molecules involved in the regulation of many physiological and stress-related processes in plants, including root growth, senescence, fruit ripening, wounding, water deficit, and pathogen attack [1–8].

Recently, the underlying mechanism of JA signaling networks during stress responses and development including cooperation between various hormone responses were addressed [9]. Jasmonate ZIM-domain (JAZ) proteins and their interacting partners play essential roles in orchestrating the cross talk between JA and other hormone signaling pathways including gibberellins (GAs), salicylic acid (SA), abscisic acid (ABA), auxin, and ethylene (ET) [7,10,11].

JAZ proteins belong to the TIFY family, which constitutes several plant-specific groups of proteins with various functions [12]. The TIFY family consists of four subgroups including ZML, TIFY, PPD, and JAZ proteins [13]. In the absence of bioactive JA, JAZ proteins repressed the downstream gene expression by physically interacting with members of the R/B-like basic helix-loop-helix (bHLH) transcription factors (TFs), including MYC2, MYC3, and MCY4 [14–16]. In response to stress, such as insect feeding or pathogen infection, the level of bioactive JA increases. An F-box protein CORONATINE INSENSITIVE1 (COI1) perceives JA signals and recruits JAZ proteins for ubiquitin-mediated protein degradation. As a result, R/B-like bHLH TFs are released from JAZ proteins, and the downstream genes required for JA-mediated responses are activated [17,18]. Furthermore, the function of several co-regulators of JAZ proteins were identified, such as Novel Interactor of JAZ (NINJA), TOPLESS proteins (TPL), ethylene-stabilized TFs (EIN3/EIL1), R2R3-MYB TFs (MYB21/MYB24), and DELLA proteins [19]. The NINJA protein was identified using a tandem affinity purification method, and NINJA was co-purified with TPL and the related protein TPR, which has been implicated in the regulation of auxin signaling pathways [20,21]. EIN3 and EIL1 are TFs that respond to ethylene and activate a subset of genes involved in pathogen defense and developmental regulation [22]. The activity of EIN3 and EIL1 can be regulated by JA through binding with JAZ proteins [23], indicating that JAZ degradation by JA signaling is required for full activation of these TFs. The R2R3 MYB-type TFs, MYB21 and MYB24, have also been identified as JAZ-interacting proteins [24]. The *myb21* mutant showed reduced fertility, and *myb24* exacerbated this phenotype, while overexpression of *MYB21* partially restores the sterility phenotype of *coi1* [24,25]. These results suggested that MYB21 and MYB24 may be implicated in JA-mediated male fertility. DELLA proteins function as essential regulators of GA signaling, and they are responsible for repressing the expression of GA-induced genes in the absence of hormone [26]. Repressor of gal-3 (RGA), a DELLA protein, was identified as a JAZ1 binding protein, and further analysis revealed that RGA and MYC2 compete for binding to JAZ1, indicating that DELLA may diminish the interaction between JAZ and MYC2 and thus relieve JAZ repression of JA-inducible genes [27]. These studies indicate that JAZ proteins may serve as transcriptional repressors in JA signaling pathways and act as hubs in an extensive signaling network, influencing various hormone pathways and aspects of plant growth. Characterizing hormonal cross talk is essential to understand how plants react to a specific stress. To accomplish this task, we examined the functions of JAZ proteins and elucidated their roles in cross-talk regulation.

JAZ proteins are found in many plant species, and the number of isoforms within this family is variable among different species. Twelve, 11, 15, and 23 genes encoding JAZ family proteins have been reported in *Arabidopsis*, grape, rice, and maize [13], respectively, indicating that the JAZ family may have evolved from common ancestors and possess critical functions. In addition, the expression patterns of JAZ family genes were determined in rice and grape [28,29], but not in maize. Moreover, although the functions of many JAZ proteins have been characterized in *Arabidopsis* [17,30,31], rice [28,32], tobacco [33], and *Glycine soja* [34], few functional studies are available on *JAZ* genes in maize.

In this report, we provide information on the phylogeny and expression pattern of *ZmJAZ* family genes. *ZmJAZ14* was identified as a tassel and endosperm-specific *JAZ* gene and its coding protein is located in the nucleus, indicating that it may function as a transcriptional regulator. The expression of *ZmJAZ14* in response to various phytohormone stimuli and abiotic stresses was investigated using quantitative reverse transcription-PCR (qRT-PCR). In addition, we found that overexpression of *ZmJAZ14* in *Arabidopsis* enhanced plant tolerance to JA and ABA treatments, as well as polyethylene glycol (PEG) stress, and promoted growth under GA stimulus. Moreover, ZmJAZ14 interacted with a subset of TFs in *Arabidopsis*, and the expression levels of marker genes involved in JA, ABA, and GA signaling pathways were altered. Taken together, these results supported the essential role of ZmJAZ14 as a regulator of cross talk among the JA, ABA, and GA signaling pathways.

## Materials and Methods

### Plant materials, growth conditions, and plants treatments

The plants of maize (*Zea mays* L.) inbred line B73 were grown in the experimental station of Biotechnology Research Institute, Chinese Academy of Agricultural Sciences, Hebei province, China. For expression analysis of *ZmJAZ14* and *ZmJAZ4*, root, stalk, leaf, and apex samples were collected from the flare opening stage, silks and tassels were collected at the flowing period, as well as embryos and endosperms were sampled on 10, 15, 20, and 25 days after pollination. For phytohormone and abiotic stress treatments, the maize seedlings with three leaves were used. The seedlings were planted in vermiculite and transplanted into water 2 h before treatments to balance the variable conditions. The seedlings were then subjected to water (control), 250 mM NaCl, 20% PEG, 100 μM JA, 100 μM ABA, 100 μM GA, and 100 μM SA. Samples of shoots and roots were separated and taken as three biological replicates at 0, 0.5, 1, 3, 6, 9, and 24 h after treatment.

*Arabidopsis thaliana* (Col-0) seeds were chilled at 4°C for 3 days. Seeds were then sterilized by 75% ethanol containing 0.02% Tween 20 for 5 min and washed with distilled water four times. Seeds were then sown on 0.5× MS medium and transferred to a culture room with a 16 h/8 h light/dark photoperiod at 22°C.

To examine phytohormones and abiotic responses of transgenic *Arabidopsis* lines, after 7 days of growth on 0.5× MS medium, the seedlings were transferred to 0.5× MS solid medium with or without 10 mM NaHCO_3_, 15% PEG, 25 μM JA, 10 μM GA, and 1 μM ABA. The phenotypes were observed 7 days after transplantation and were vertically grown in a chamber. WT and transgenic lines were grown on the same plate to control for plate-to-plate variation. The lengths of roots were measured using ImageJ software. Statistical calculations were performed using SPSS software.

### Sequence alignment and phylogenetic tree construction

The TIFY family proteins were identified in maize by searching the TF database (http://plntfdb.bio.uni-potsdam.de/v3.0). The deduced protein sequences of ZmTIFY proteins were aligned using ClustalX 2.0.5 program. The phylogenetic tree was constructed using the neighbor-joining method in MEGA 4.0 software. The ZmTIFY proteins and their accession numbers used for phylogenetic tree construction are as follows: ZmJAZ1 (GRMZM2G343157), ZmJAZ2 (GRMZM2G445634), ZmJAZ3 (GRMZM2G117513), ZmJAZ4 (GRMZM2G024680), ZmJAZ5 (GRMZM2G145412), ZmJAZ6 (GRMZM2G145458), ZmJAZ7 (GRMZM2G382794), ZmJAZ8 (GRMZM2G086920), ZmJAZ9 (GRMZM2G145407), ZmJAZ10 (GRMZM2G171830), ZmJAZ11 (GRMZM2G005954), ZmJAZ12 (GRMZM2G101769), ZmJAZ13 (GRMZM2G151519), ZmJAZ14 (GRMZM2G064775), ZmJAZ15 (GRMZM2G173596), ZmJAZ16 (GRMZM2G338829), ZmJAZ17 (GRMZM2G126507), ZmJAZ18 (GRMZM2G116614), ZmJAZ19 (GRMZM2G066020), ZmJAZ20 (GRMZM2G089736), ZmJAZ21 (GRMZM2G036351), ZmJAZ22 (GRMZM2G036288), ZmJAZ23 (GRMZM2G143402).

### Subcellular localization

For subcellular localization experiments, the coding region of *ZmJAZ14* was amplified with the primers JAZ14GFP-U and JAZ14GFP-L, and the resulting fragment was cloned into the *Xho*I-*Xba*I sites of plant expression vector pRTL2GFP [35] to generate the transient expression vector pRTL2GFP-*ZmJAZ14*, in which ZmJAZ14 was C-terminal in-frame fused with GFP. The plasmids pRTL2GFP and pRTL2GFP-*ZmJAZ14* were transformed into maize mesophyll protoplasts as described previously [36]. Hoechst 33342 was used at a final concentration of 5 μg/mL to stain the nucleus. After 16 h of incubation, the GFP and Hoechst fluorescence was observed under a confocal microscope (LSM700; Carl Zeiss, Wetzlar, Germany).

### Transcriptional activation analysis

To examine the self-activation of ZmJAZ14 in yeast, the coding region of *ZmJAZ14* was amplified using the primers JAZ14BD-U and JAZ14BD-L, and the resulting fragment was cloned into pGBK-T7 to generate pGBK-*ZmJAZ14*. To examine the self-activation ability of ZmJAZ14, pGBK-*ZmJAZ14* and pGAD-T7 were co-transformed into yeast strain AH109 using the LiAc-PEG method. The transformed colonies were selected on SD glucose medium without Leu and Trp (DDO), and the self-activation tests were surveyed on selective medium lacking Leu, Trp, Ade, and His (QDO). The assay was performed in duplicates and a similar result was observed.

### Construction of the plant expression vector and generation of transgenic plants

The maize ubiquitin promoter was amplified using the primers UBI-U and UBI-L. The PCR fragment was digested with *Eco*RV and *Bam*HI and introduced into the *Eco*RI-*Bam*HI site (the *Eco*RI site was blunted by T4-DNA polymerase) of pCAMBIA-3301 to generate the binary vector pCAMBIA-UBI. To construct the overexpression vector pCAMBIA-UBI-*ZmJAZ14*, the coding region of *ZmJAZ14* was amplified with the primers JAZ14-U and JAZ14-L, and the resulting fragment was cloned into the *Bam*HI-*Bst*EII site of pCAMBIA-UBI to generate pCAMBIA-UBI-*ZmJAZ14*. This construct was transformed into *Arabidopsis* using the floral dip method. The transgenic plants were screened on 0.5× MS medium containing 20 mg/L phosphinothricin. The homozygous lines were generated by self-fertilization.

### qRT-PCR analysis

RNA was extracted using Trizol reagent (TaKaRa, Otsu, Japan) following the manufacturer’s instructions and then treated with RNase-free DNase I (Promega, Madison, WI) to remove genomic DNA. First strand cDNA was synthesized using M-MLV reverse transcriptase (Thermo Fisher Scientific, Waltham, MA). For the spatial and temporal expression pattern in various maize tissues and the expression profiles under phytohormone and abiotic stimuli, the gene specific primers for *ZmJAZ14*, *ZmJAZ4*, *ZmJAZ12* and *ZmJAZ20* were applied (S1 Table), and the expression level of *ZmActin1* was used as an internal control. For expression analysis of *Arabidopsis* genes, the total RNA was extracted from seedlings and the cDNA was synthesized as described previously. Specific primers for qRT-PCR analysis of *Arabidopsis* genes were listed in S1 Table. The expression of *AtActin2* (At3g18780) was used as an internal control. qRT-PCR was performed using ABI 7500 system (Applied Biosystems, Foster City, CA), as described previously [35]. The PCR conditions included an initial denaturation at 95°C for 30 s, followed by 40 cycles of amplification for 5 s at 95°C and 34 s at 60°C. The melting-curve analysis was applied, and the qPCR product was also analyzed by electrophoresis and sequenced to verify the specific amplification. Data were analyzed using the ABI 7500 software (version 2.0.5) using the ΔΔCT method (Applied Biosystems). Three biological replicates were used and three technical replicates were performed for each biological replicate.

### Yeast two-hybrid assays

To examine the interaction between ZmJAZ14 and TFs in *Arabidopsis*, a GAL-4 based yeast two-hybrid system was used. The TFs in *Arabidopsis* were amplified using primers listed in S1 Table and cloned into the corresponding sites of pGAD-T7. The resulting plasmids were then integrated into yeast AH109 together with pGBK-*ZmJAZ14*. The interaction tests were surveyed as described in previous transcriptional activation analyses.

### Bimolecular fluorescence complementation analysis (BiFC)

The coding region of *ZmJAZ14*, *MYC2*, *MYC3*, *MYB21 and NINJA* were amplified with gene specific primers (S1 Table) and cloned into pUC-SPYNE and pUC-SPYCE vectors to generate constructs for BiFC [37]. The plasmids were transformed into maize mesophyll protoplasts as described previously [36]. After incubation for 16 h, the YFP and chlorophyll fluorescence was observed under a confocal microscope (LSM700; Carl Zeiss, Wetzlar, Germany).

### Ethics statement

The maize plants (inbred line B73) were grown in the experimental station of Biotechnology Research Institute, Chinese Academy of Agricultural Sciences, Hebei province, China. Since the maize inbred line B73 is a common non-transgenic maize variety and the field studies did not involve endangered or protected species, we had permission from the Biotechnology Research Institute, Chinese Academy of Agricultural Sciences, Hebei province, China to conduct this study.

## Results

### Phylogenetic and expression analysis of the TIFY family in maize

**Fig 1:**
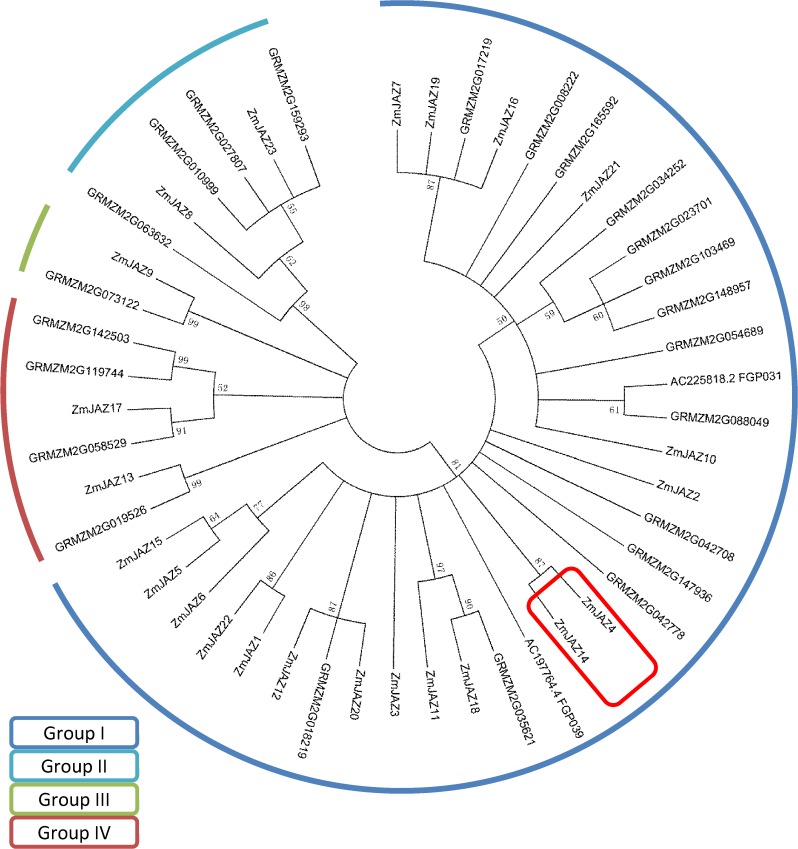
Neighbor-joining phylogenetic tree of ZmJAZ proteins. The phylogenetic tree was constructed with the amino acid sequences of JAZ proteins from maize using the neighbor-joining method. The ZmJAZ proteins were classified into four subgroups, which were identified based on different colors.

The TIFY family can be classified into four subfamilies including TIFY, JAZ, PPD, and ZML. In the present study, 48 members containing the TIFY domain were identified in maize by searching the TF database (http://plntfdb.bio.uni-potsdam.de/v3.0). ZmJAZ proteins were divided into four groups based on phylogenetic analysis (Fig. 1), and many of these proteins were sister pairs due to duplication of the chromosomal blocks.

To examine the expression profiles of the *JAZ* genes in maize, the Affymetrix maize genome microarray was applied to detect the expression levels of the *JAZ* genes in different organs [38], including vegetative tissues such as root, stalk, leaf, and apex, and reproductive organs such as silk, tassel, and seeds at 10, 15, 20, and 25 days after pollination (S1 Fig.). Ten *JAZ* genes were mapped in the microarray, which were named *ZmJAZ3*, *ZmJAZ4*, *ZmJAZ8*, *ZmJAZ11*, *ZmJAZ12*, *ZmJAZ13*, *ZmJAZ14*, *ZmJAZ17*, *ZmJAZ20*, and *ZmJAZ23* in a previous study by Bai et al. [13]. We found that the expression patterns of *ZmJAZ* genes varied significantly, as *ZmJAZ13* and *ZmJAZ23* were constitutively expressed in every tissue, *ZmJAZ* members of 3, 8, 12, 13, 17, and 20 showed a high expression level in vegetative tissues, and *ZmJAZ4* and *ZmJAZ14* specifically accumulated in the endosperm.

### *ZmJAZ14* was specifically expressed in the tassel and developing endosperm and serves as a transcriptional regulator

**Fig 2:**
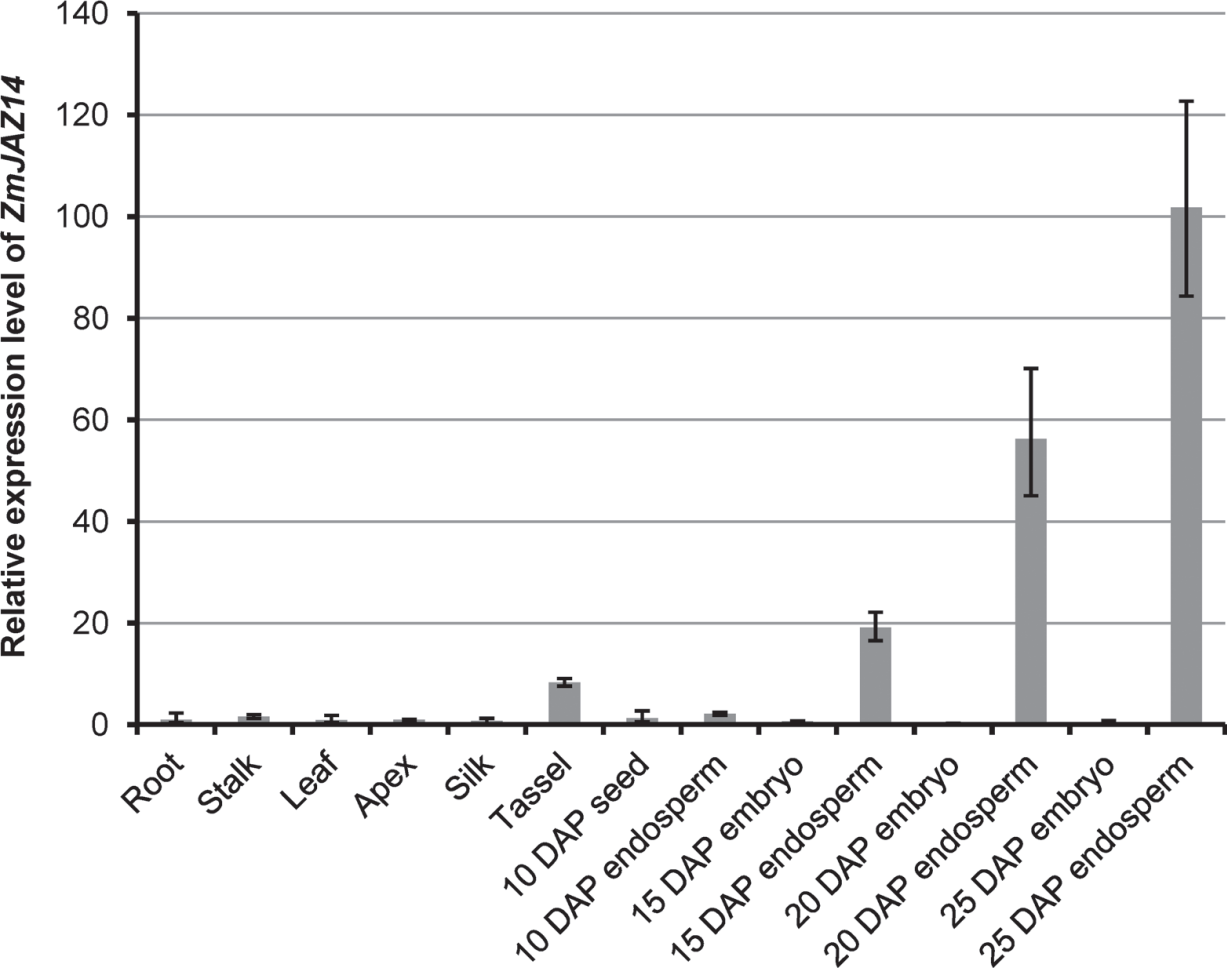
Expression levels of *ZmJAZ14* in different maize organs. The relative expression level of *ZmJAZ14* was normalized with *ZmActin1*. Data from qRT-PCR were analyzed according to the 2^-ΔΔCt^ method. The error bars indicate standard deviations.

**Fig 3:**
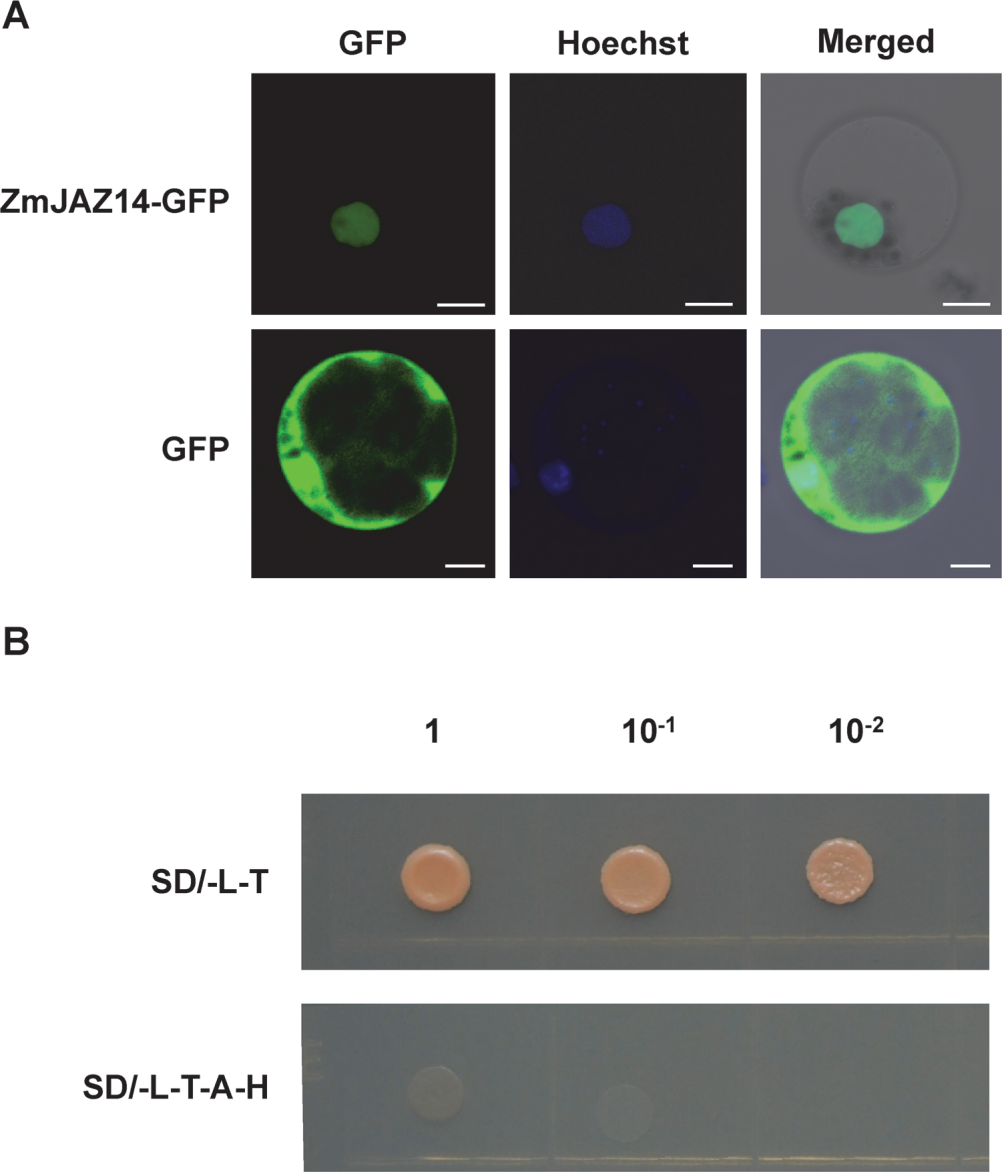
Subcellular localization and transcriptional activation analysis of ZmJAZ14. The coding region of *ZmJAZ14* was C-terminally fused with GFP and transiently expressed in maize mesophyll protoplasts. The GFP signal of ZmJAZ14-GFP was detected in the nucleus, and the cytoplasmic localization of GFP was used as a control (A). The nucleus was stained by Hoechst, and bars represent 10 μm. The transcriptional activation activity of ZmJAZ14 was analyzed in yeast (B). The plasmid pGBK-*ZmJAZ14* and pGAD-T7 were co-transformed into the yeast strain AH109. Yeast transformants were diluted and spotted onto control medium (SD/-L-T, lacking Leu and Trp) or selective medium (SD-L-T-A-H, lacking Leu, Trp, Ade and His).

The JAZ member ZmJAZ4 and ZmJAZ14 were classified in group I; thus, they may be functionally redundant. The expression pattern may be useful to infer the functions of *ZmJAZ4* and *ZmJAZ14*. Therefore, qRT-PCR was applied to verify the mRNA accumulation profiles of *ZmJAZ4* (S2 Fig.) and *ZmJAZ14* (Fig. 2) in various organs and developing seeds using maize *Actin1* as an internal control. Notably, *ZmJAZ4* and *ZmJAZ14* were both predominantly expressed in tassel and endosperm, and the intensity gradually increased during endosperm development. This result suggests that *ZmJAZ4* and *ZmJAZ14* may be redundant in functions, and *ZmJAZ14* was selected for further study. The subcellular localization of ZmJAZ14 may help to understand the role of this gene in maize plants. Therefore, the coding region was fused in-frame to the N-terminus of GFP controlled by a duplicated 35S promoter. The vector was transferred and expressed transiently in maize protoplasts. The ZmJAZ14-GFP fusion protein was localized to the nucleus, while GFP was observed in both the cytosol and nucleus (Fig. 3A).

The transcriptional activation activity of ZmJAZ14 was analyzed using the yeast two-hybridization system. The coding region of ZmJAZ14 was cloned into the pGBK-T7 vector fused to the GAL4 DNA-binding domain, and the resultant plasmid was co-transformed into yeast with empty pGAD-T7 vector to examine the self-activating ability of ZmJAZ14. Based on these results, ZmJAZ14 possessed no transcriptional activity (Fig. 3B). Since ZmJAZ14 localized to the nucleus and no activating ability was detected, ZmJAZ14 likely functions as a transcriptional regulator.

### ZmJAZ14 may be involved in JA, ABA, and GA signaling pathways

**Fig 4:**
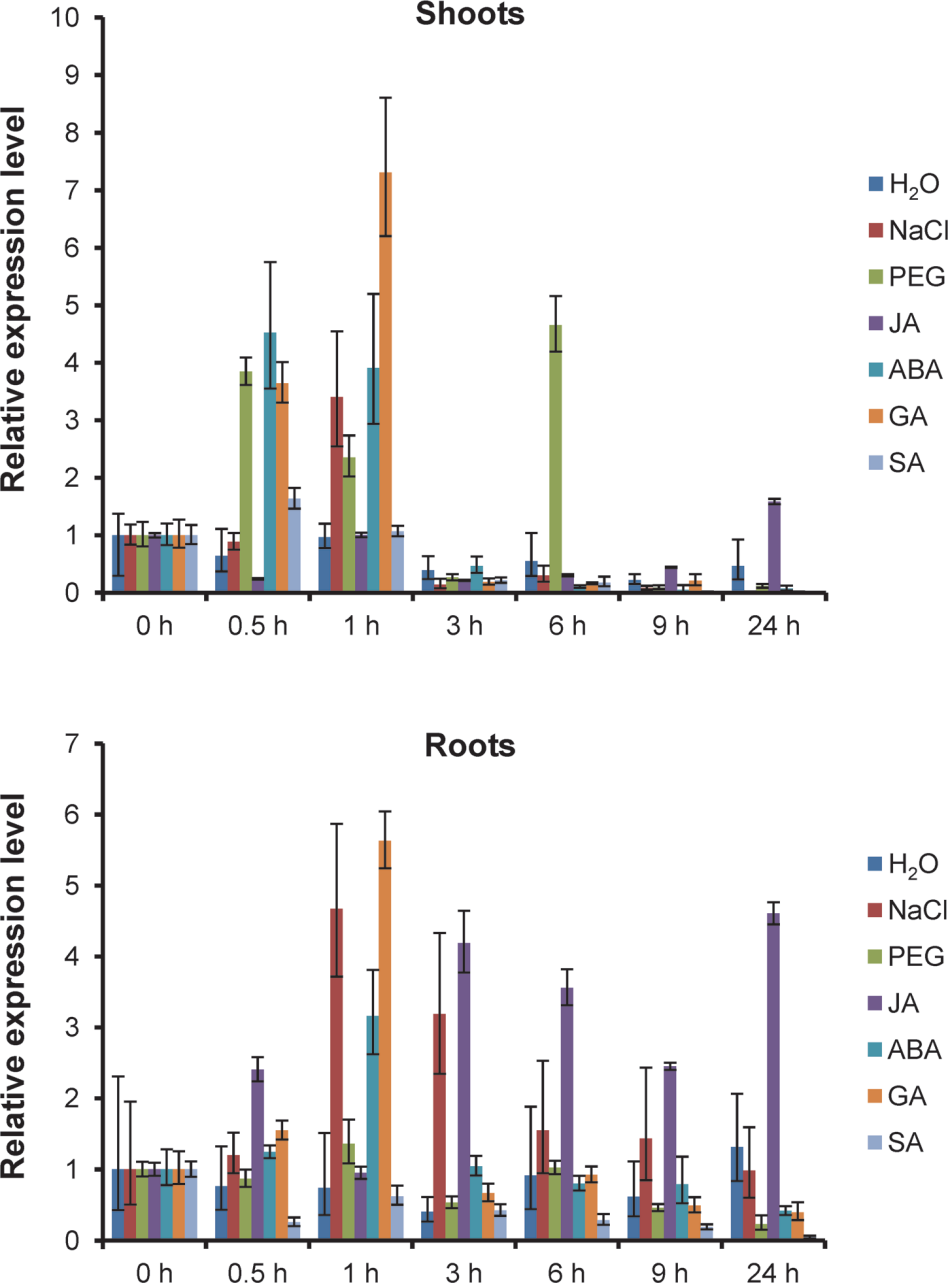
Expression of *ZmJAZ14* treated with NaCl, PEG, JA, ABA, GA, and SA in maize seedlings at the three-leaf stage. The maize seedlings were treated with water (control), 250 mM NaCl, 20% PEG, 100 μM JA, 100 μM ABA, 100 μM GA, and 100 μM SA. The shoots and roots were separated and harvested. Relative mRNA abundance of *ZmJAZ14* was normalized with the *ZmActin1* gene. Data from real-time RT-PCR experiments were analyzed according to the 2^-ΔΔCt^ method and the error bars indicate standard deviations.

Numerous treatments were applied to inspect the expression of *ZmJAZ14* in response to phytohormones or abiotic stresses. The transcripts of *ZmJAZ14* were significantly induced in shoots by PEG, high salt, ABA, and GA treatment, and were upregulated in response to high salt, JA, ABA, and GA treatment in roots. In contrast, the accumulation of *ZmJAZ14* was repressed under SA treatment in both shoots and roots (Fig. 4). To compare the inducibility of *ZmJAZ14* with other *JAZ* genes, the expression pattern of *ZmJAZ12* and *ZmJAZ20* under various stimuli were examined (S3 Fig.). The expression of *ZmJAZ12* and *ZmJAZ20* were stimulated quickly by NaCl, ABA and GA in shoots, while that were induced by NaCl and PEG in roots. Compare to *ZmJAZ14*, *ZmJAZ12* and *ZmJAZ20* were not induced by JA in both shoots and roots, and their expressions remain unchanged in response to ABA and GA in roots. Since *ZmJAZ14* responded to ABA and GA during the early stages of treatment and responded to JA in roots during the late stages of treatment, *ZmJAZ14* may be associated with different aspects of the ABA, GA, and JA signaling pathways.

### Overexpression of *ZmJAZ14* in *Arabidopsis* altered the responses to various phytohormone stimuli and abiotic stresses

**Fig 5:**
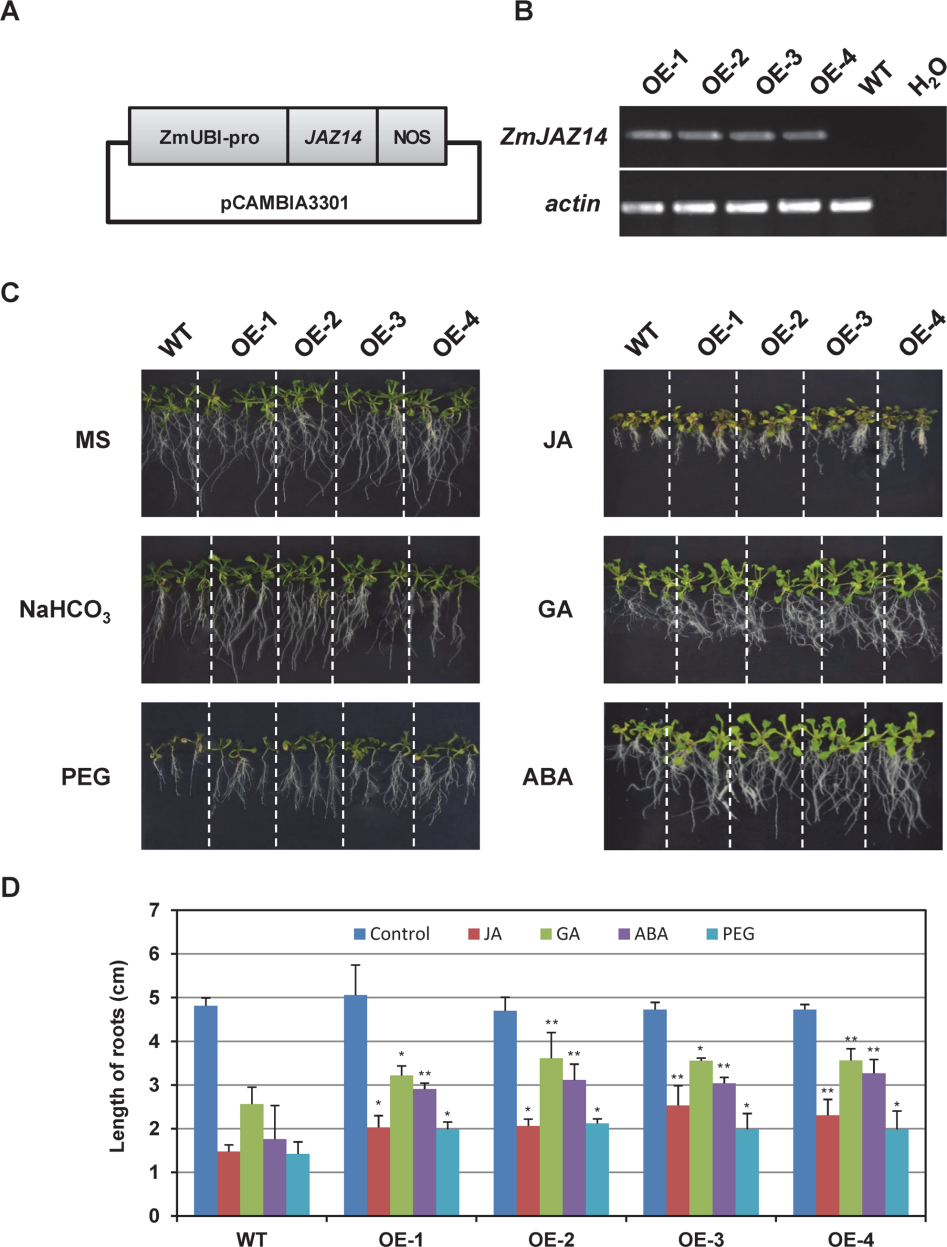
*ZmJAZ14* overexpression (OE) plants showed enhanced stress and phytohormone tolerance. (A). RT-PCR verifying the expression of *ZmJAZ14* in transgenic plant lines; *AtActin2* was used as an internal control (B). Phenotypes of *ZmJAZ14* transgenic (OE1-OE4) and WT lines under 10 mM NaHCO_3_, 15% PEG, 25 μM JA, 10 μM GA, and 1 μM ABA conditions. (C). Root length of WT and OE lines under different stress conditions. Error bars indicate standard deviations. Asterisks, * and **, refer to significant differences (*P*<0.05 and *P*<0.01).

To characterize further the physiological function of *ZmJAZ14*, overexpressing transgenic *Arabidopsis* plants were generated, which expressed *ZmJAZ14* under control of a maize ubiquitin promoter (Fig. 5A). The T_0_ transformants were grown in a greenhouse to acquire T_1_ progeny for genetic analysis. Four transgenic lines with a 3:1 segregation pattern were selected for further analysis. The RT-PCR revealed that exogenous *ZmJAZ14* was successfully expressed in T_3_ homozygous transgenic plants (Fig. 5B). The phenotype of the transgenic lines and wild type (WT) under different phytohormone and stress conditions was investigated to characterize the function of *ZmJAZ14*. Although no differences were detected between *ZmJAZ14*-overexpression (OE) and WT lines grown in Murashige & Skoog (MS) medium alone, the growth trend differed between OE and WT lines when phytohormones and 15% PEG were added to MS medium. The transgenic plants grew better and formed relatively more lateral roots than the WT under 25 μM JA, 10 μM GA, and 1 μM ABA treatment. In addition, the OE lines had better PEG stress resistance than the WT, as the root growth of OE plants was significantly better than that of WT when grown in MS with 15% PEG (Fig. 5C and D).

### Expression analysis of JA, ABA, and GA signaling pathway-related genes between the transgenic and WT plants

**Fig 6:**
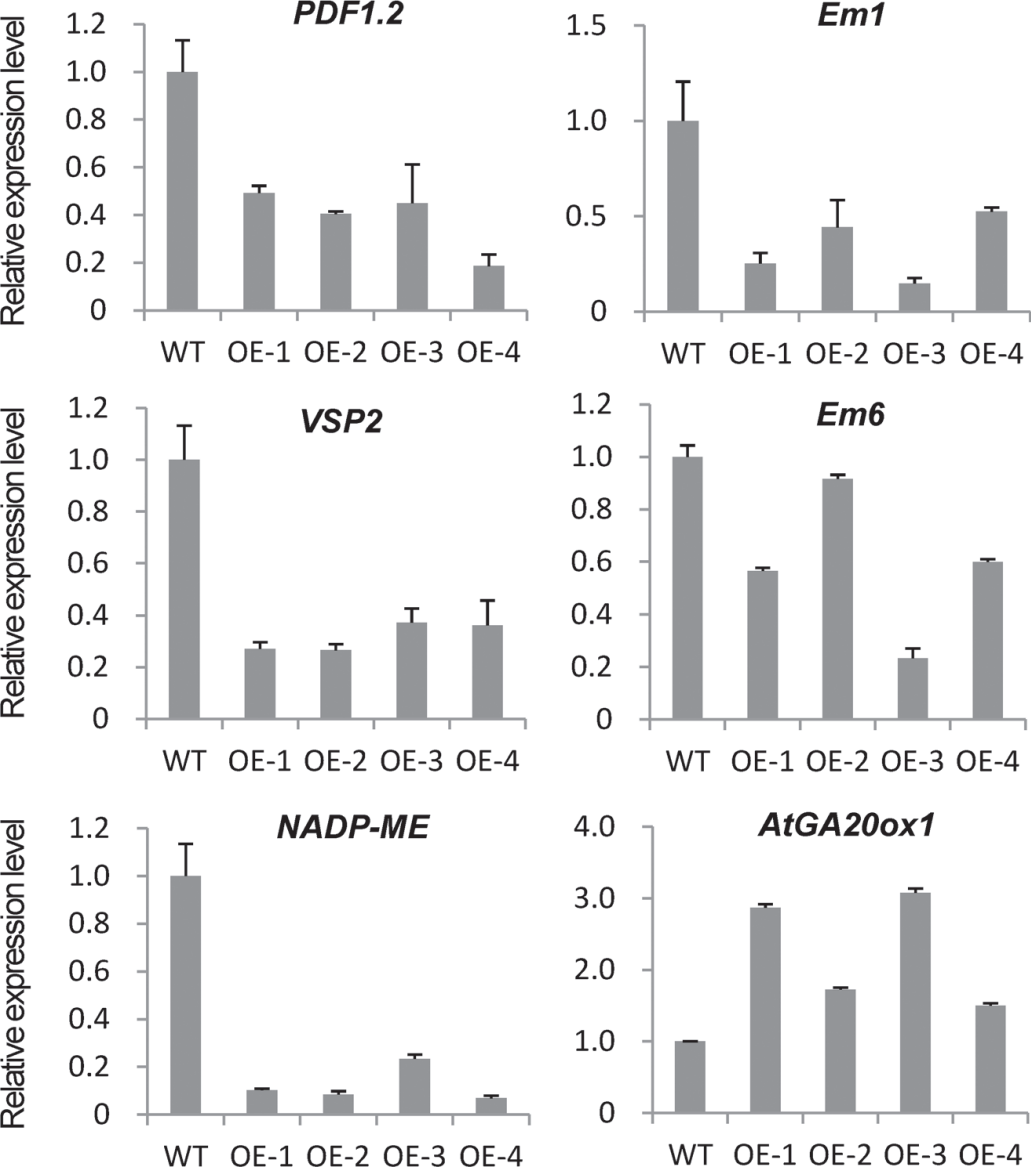
Expression levels of genes involved in JA, ABA, and GA signaling pathways were altered in *ZmJAZ14* overexpression (OE) lines. The expression of marker genes in JA, ABA, and GA signaling pathways were analyzed in the wild-type and *ZmJAZ* OE lines. Seedlings were harvested after 1 week of treatments. Relative gene expression was normalized using *AtActin2*. The error bars indicate standard deviations.

To examine the regulation mechanism of *ZmJAZ14*, the expression of genes associated with JA, ABA and GA signaling pathways were examined, including *PDF1*.*2* (*Plant defensin 1*.*2*), *VSP2* (*Vegetative storage protein*) and *NADP-ME* (*NADP-malic enzymes*) for JA, *Em1* and *Em6* (*group I late embryogenesis abundant genes*) for ABA, and *AtGA20ox1* (*GA 20-oxidase enzyme*) for GA. The results showed that the expression levels of *PDF1*.*2*, *VSP2*, *NADP-ME*, *Em1* and *Em6* were significantly down-regulated in the OE lines, whereas the accumulation of *AtGA20ox1* significantly increased (Fig. 6). In summary, overexpression of *ZmJAZ14* inhibited the transcription of JA- and ABA-inducible genes and promoted the accumulation of GA-inducible genes, which is consistent with the JA-, ABA-, and GA-related phenotype observed in *ZmJAZ14*-OE plants.

### ZmJAZ14 interacts with typical TFs in *Arabidopsis*

**Fig 7:**
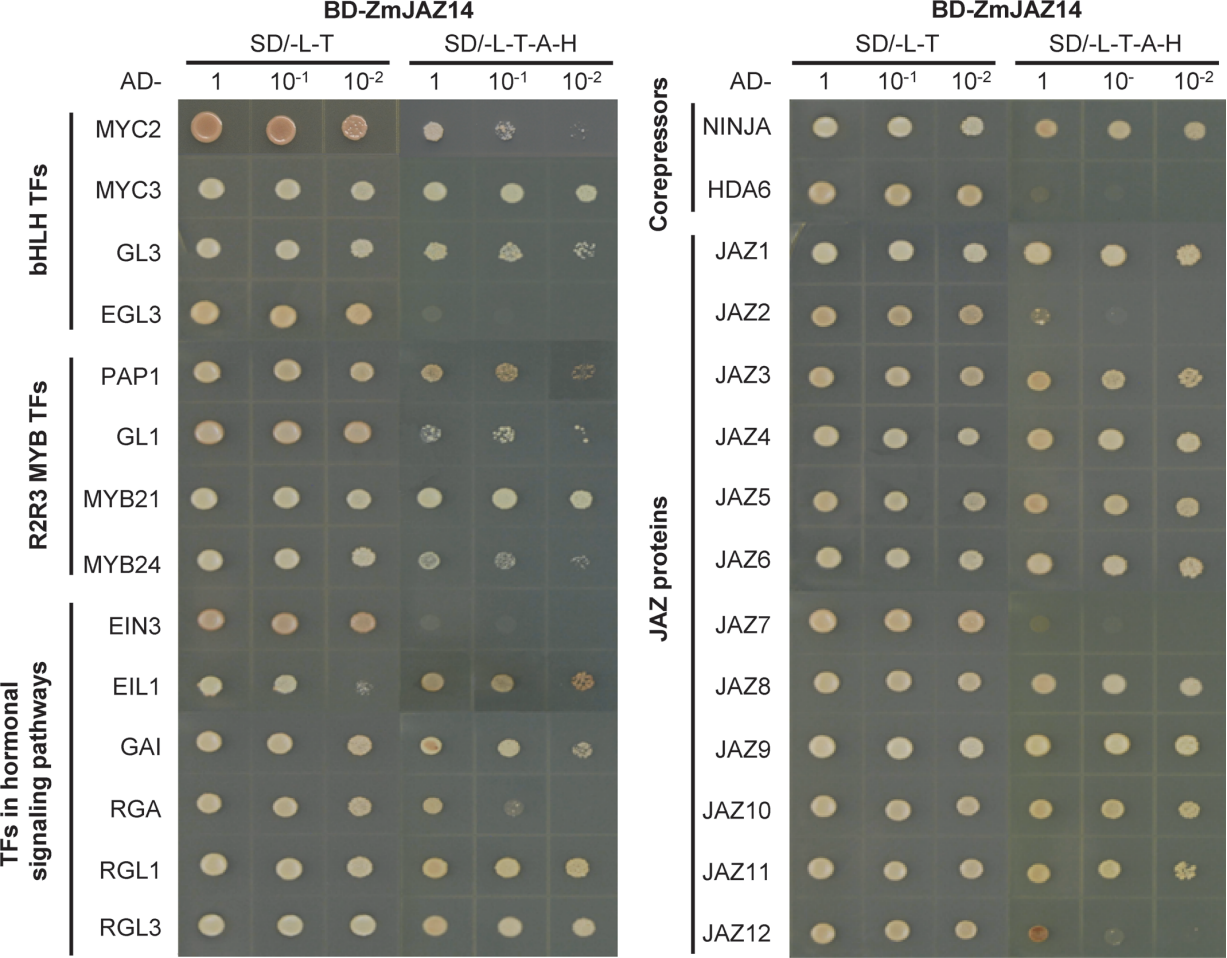
ZmJAZ14 interacted with transcription factors (TFs) involved in various signaling pathways in *Arabidopsis*. The yeast two-hybrid system was applied to verify the interaction between ZmJAZ14 and functionally characterized TFs in *Arabidopsis*. The transformants were grown on SD minimal medium (SD/-L-T) lacking leucine and tryptophan and (SD-L-T-A-H) lacking leucine, tryptophan, adenine, and histidine. Cell concentration was adjusted to OD_600_ = 1 and serial dilutions were spotted on plates and grown for 4 days at 28°C.

**Fig 8:**
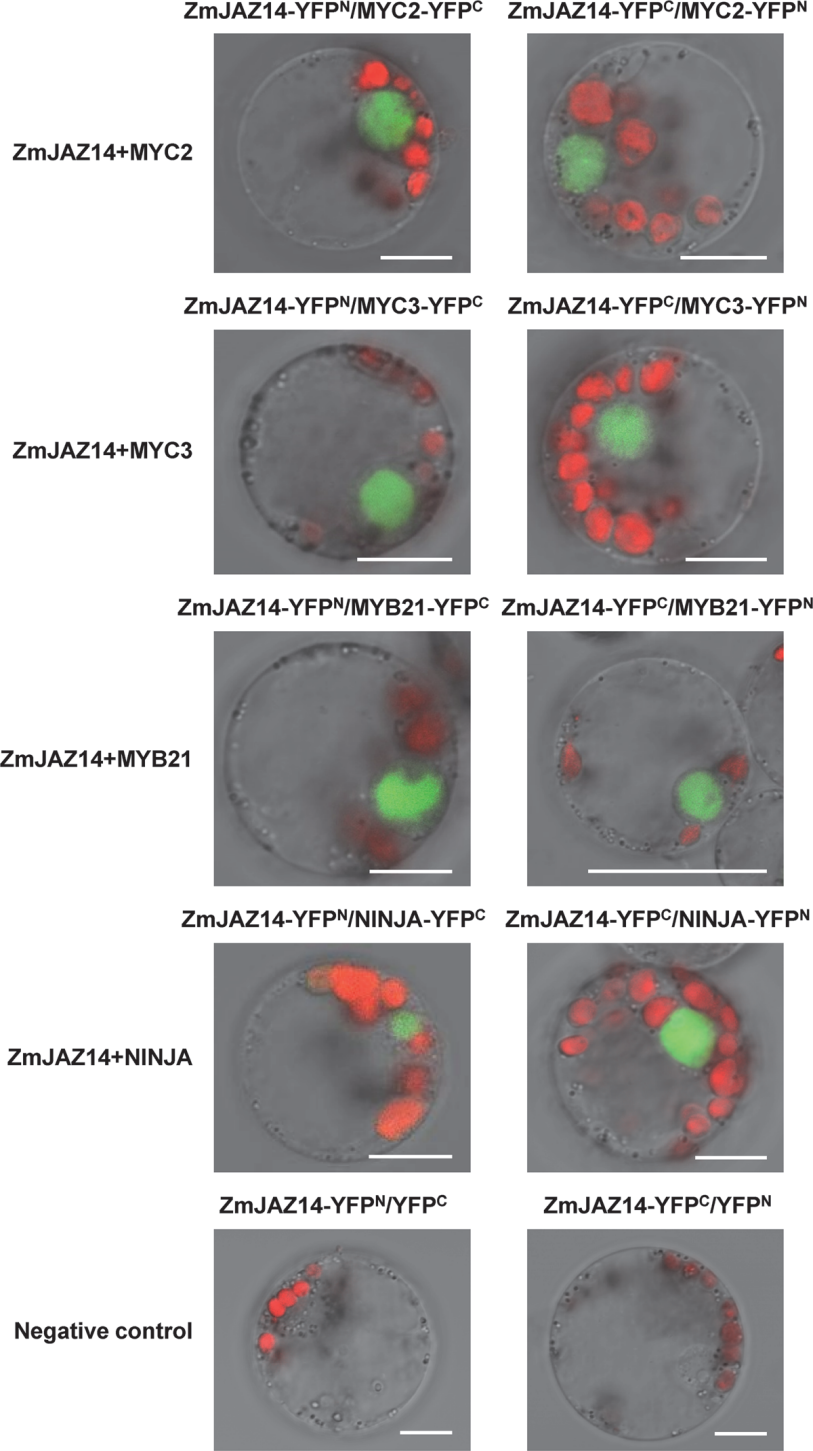
BiFC assay to verify interactions between ZmJAZ14 and transcription factors (TFs) from *Arabidopsis*. The coding regions of *ZmJAZ14* and *TFs* were fused to the N-terminal or C-terminal fragment of YFP. Construct pairs indicated on the top of panels were co-expressed in maize protoplasts. The YFP signal is indicated in green and the chlorophyll autofluorescence is indicated in red. The merged images of fluorescence and bright field are shown. Bars represent 10 μm.

As a repressor, ZmJAZ14 contains an EAR-like motif, which is known to function in gene repression by recruiting co-repressors such as TPL and histone-modifying enzymes to inhibit transcription. Therefore, a set of typical TFs implicated in JA and other signaling pathways was cloned and examined for the interaction with ZmJAZ14 by yeast two-hybrid assays (Fig. 7). ZmJAZ14 was found to interact with MYC2, MYC3, and GL3 as bHLH TFs, and PAP1, GL1, MYB21, and MYB24 as R2R3 MYB TFs, and EIL1, GAI, RGA, RGL1, and RGL3 as TFs involved in other hormonal signaling pathways, as well as NINJA corepressors. Moreover,ZmJAZ14 could bind to most of the JAZ proteins from *Arabidopsis*, except AtJAZ7. The interactions of ZmJAZ14 with MYC2, MYC3, MYB21 and NINJA were confirmed further by BiFC (Fig. 8). The results revealed that ZmJAZ14 could interact with the majority of factors and thus must be involved in several biological processes.

## Discussion

JAs are a type of phytohormone that regulate the switch between growth and stress-resistance responses, whereas GAs are a class of hormones required for various growth processes such as germination, hypocotyl elongation, and flower development. Therefore, JAs function in defense, while GA plays roles in growth promotion. Examining why these two hormones act in an antagonistic fashion and how this system functions is important. Identification and characterization of JAZ proteins and their interactors may shed light on the mechanism and reveal how plants modulate these regulatory responses. Since the expression of *ZmJAZ14* was altered under various phytohormone treatments, *ZmJAZ14* may serve as a node for the cross talk among different phytohormone signaling pathways.

### Identification and expression analysis of *JAZ* family genes in maize

Maize is not only an important commercial crop, but also a model plant for genetics and evolutionary studies. The kernel quality and stress resistance during reproductive and grain-filling stages are the major factors that influence corn yield. Therefore, identifying functional genes essential for growth and stress-resistance regulation at the seed developing stages is useful for breeding maize varieties with high and stable yield. Recently, many genes have been characterized that play important roles during maize development. JAZ proteins constitute a major TF family in plants and play essential roles in regulating growth and development. Since the identification of JAZ proteins in *Arabidopsis*, several JAZ family proteins have been identified and characterized in the model plants *Arabidopsis* [12,13], grapes [29], and rice [28], whereas few JAZ TFs have been studied in maize. Since the genome-sequencing of maize inbred line B73 has been completed, many TF families were identified based on bioinformatic analysis, including the ARF [39], bZIP [40], and MADS-box [41] families. However, few systematic studies have examined the JAZ family in maize, excluding 23 genes encoding ZmJAZ proteins [13]. In the present study, 48 *ZmTIFY* genes were obtained by searching the TF database (http://plntfdb.bio.uni-potsdam.de/v3.0), and their expression patterns were determined by analyzing the microarray data. In addition, the majority of ZmJAZs were present as sister pairs and share high similarity between paralogs, and the duplication of *ZmJAZ* genes may be associated with the chromosomal block duplications [42]. ZmJAZ4 and ZmJAZ14 share 70% identity and form a pair of paralogs. Expression analysis showed that *ZmJAZ4* and *ZmJAZ14* both accumulated specifically in developing endosperms. This result indicated that *ZmJAZ4* and *ZmJAZ14* may function redundantly and play essential roles in the maturation of endosperm.

### ZmJAZ14 serves as a transcriptional regulator and may be involved in multiple developmental processes in maize

The nuclear localization of ZmJAZ14 may contribute to increase our understanding of ZmJAZ14. The nuclear localization signal (NLS) of JAZ proteins had been reported to be ambiguous, though many studies have demonstrated that JAZ is localized in the nucleus and functions as a transcriptional regulator [43]. Notably, the NLS is different from the reported R/H/KX(2–5)PY consensus sequence in the JAZ family [44]. Since the Jaz domain are implicated in the interaction with other transcription factors, the extended Jaz domain of ZmJAZ14 may also play a role in targeting the protein to the nucleus [13].

It is also found that ZmJAZ14 does not possess transcriptional activation ability (Fig. 3B). Although the JAZ family proteins do not contain a DNA-binding domain, they were considered transcriptional regulators since they could regulate the expression of downstream genes by interacting with various TFs. Therefore, the nuclear localization of ZmJAZ14 and its lack of transcription activity are consistent with an expression regulator role for this protein.

Analysis of a 2000-bp sequence upstream of the *ZmJAZ14* gene identified many *cis*-elements required for meristem, seed, and endosperm-specific expression. In addition, ABA-responsive elements were found in the promoter region (S2 Table). It is known that ABA levels increase during seed maturation. Hence, an increase of *ZmJAZ14* expression associated with endosperm development may contribute to ABA regulation. The expression of *JAZ* genes under phytohormone or stress is essential to examine the underlying mechanism. *JAZ* genes have been reported to be induced by 1-naphthaleneacetic acid (NAA) [43], ethylene [45], wounding [33], herbivores [46], salt, and alkali [47]. Hormone and stress treatments played crucial roles in the induction of *ZmJAZ14* expression in maize seedlings. In shoots, this gene was induced by salt, PEG, GA, and ABA and salt, JA, GA, and ABA in roots. In contrast, the transcript level of *ZmJAZ14* tended to be down-regulated both in shoots and roots under SA treatment, showing antagonism between the defense hormones SA and JA. Notably, the induction of *ZmJAZ14* by JA was delayed as compared to other hormones, and its induction level by JA was relatively weaker than those by NaCl, PEG, ABA, and GA in shoots (Fig. 4). This result indicates that *ZmJAZ14* may be important for late response to JA treatments and associated with different aspects of the ABA, GA, and JA signaling pathways. To compare the inducibility of *ZmJAZ14* with other *JAZ* genes which were highly expressed in roots and leaves of maize, the expression pattern of *ZmJAZ12* and *ZmJAZ20* under various stimuli were examined. Both *ZmJAZ12* and *ZmJAZ20* were shown to be induced quickly by NaCl, ABA, and GA in shoots and by NaCl and PEG in roots. In contrast to *ZmJAZ14*, *ZmJAZ12* and *ZmJAZ20* were not induced by JA in both shoots and roots, and their expressions remain unchanged in response to ABA and GA in roots. Taken together, these results indicate that ZmJAZ14 may play a role in hormone signaling or abiotic stress responses.

### ZmJAZ14 is involved in the cross talk between various phytohormones and stress signaling pathways by interacting with TFs in *Arabidopsis*

The physiological functions of many JAZ proteins have been determined using reverse genetic analysis [28,32,48]. Although *ZmJAZ14* was specifically expressed in tassel and endosperm, its accumulation was induced under various hormone and stress treatments in both shoots and roots. In addition, the up-regulation of *ZmJAZ14* transcripts associated with the endosperm development may be attributed to the increasing level of ABA content in seeds. These results suggested that the function of ZmJAZ14 may not only restrict to tassel and endosperm development, but also in many aspects of growth and stress responses. Moreover, overexpressing *ZmJAZ14 Arabidopsis* lines showed a stronger tolerance toward 15% PEG as well as an enhanced response to GA treatment than the WT, while they exhibited a reduced response to JA and ABA stimuli.

JAZ proteins act as JA co-receptors and transcriptional repressors in JA signaling [14–16]. To examine the function of *ZmJAZ14* in JA signaling, the root lengths were measured in the OE and WT plants under 50 μm JA treatment (Fig. 5C and D). In contrast to the WT, the transgenic lines showed impaired phenotype of JA-induced root growth inhibition, indicating that ZmJAZ14 can mediate the JA signaling pathway in *Arabidopsis*. JAZ proteins have been shown to inhibit JA responses by interacting with a wide range of TFs, including MYCs and MYBs [14–16]. MYC2 is the first identified transcription factor that regulated by JAZ proteins [17], and it functions as a positive regulator of JA-mediated inhibition of primary root growth, anthocyanin synthesis and oxidative stress tolerance, while it acts as a negative regulator of JA-mediated resistance to necrotrophic fungi [8,49]. Besides MYC2, JAZ proteins also interact with MYC3 and MYC4, which are homologs of MYC2 [15,50,51]. In contrast to MYC2, MYC3 and MYC4 regulate different subsets of JA responses, as they play a weak role in mediating JA-dependent inhibition of primary root growth, while they are important for JA-dependent defense. Moreover, a number of TFs have been identified as targets of JAZ proteins and each of them control specific downstream processes [9]. Therefore, it can be postulated that each JAZ protein may have its specificity in binding TFs and regulate specific subset of JA responses. In our study, the interactions of ZmJAZ14 with TFs from *Arabidopsis* were investigated to further explain the function of ZmJAZ14. Notably, ZmJAZ14 interacted with PAP1, MYB21 and GAI, which are shown interacted with AtJAZ1 but not interacted with most of other JAZ proteins in *Arabidopsis* [9], indicating that ZmJAZ14 has its specificity in binding downstream TFs. Furthermore, in the transgenic *Arabidopsis* lines, the expression level of JA-upregulated genes, such as *AtVSP2* and *AtPDF1*.*2* [1,52–54], were repressed, suggesting that excess amounts of ZmJAZ14 proteins may repress the transcription of JA inducible genes by interacting with TFs in *Arabidopsis*.

In previous studies, the overexpression of JAZ proteins in *Arabidopsis* led to GA-associated phenotypes such as increased plant height as well as enhanced petiole and hypocotyl elongation. These results may suggest that the association between JAZ and mediation of GA signaling pathways [55]. To examine the effect of *ZmJAZ14* in GA signaling, root lengths of OE lines were measured (Fig. 5C and D). Compared with WT, these plants had longer roots and higher number of lateral roots under GA treatment. Furthermore, it was demonstrated that ZmJAZ14 interacted with GAI, RGA, RGL1, and RGL3, which belong to DELLA family proteins and play key roles in repressing GA signaling [56,57]. Bioactive GA facilitates the proteolysis of DELLA proteins and allows GA-responsive genes to be activated [58]. The expressions of GA-downstream genes were analyzed to evaluate the effect of overexpressing *ZmJAZ14* in GA signaling pathways. The expression level of *AtGA20ox-1*, which catalyzes the final rate-limiting steps of GA biosynthesis, was up-regulated under GA treatment [59,60]. Proteolysis of DELLA reportedly prevents the increased expression of *AtGA20ox1* [61,62], indicating that DELLA is a positive regulator of *GA20ox-1*. Taken together, our results suggest that ZmJAZ14 regulates the GA signaling pathway by interacting with DELLA proteins.

The transgenic plants overexpressing *ZmJAZ14* also showed a reduced growth repression in response to ABA stimulus than the WT. In addition, the expression levels of *Em1* and *Em6* were repressed in transgenic lines. *Em1* and *Em6* were shown to encode similar proteins and their expression levels increased under ABA stimulus [63]. Thus, overexpressing *ZmJAZ14* in *Arabidopsis* may repress the activation of ABA-inducible genes. Cross talk between the JA and ABA signaling pathways is still ambiguous since both synergistic and antagonistic interactions have been reported [64–66]. Therefore, the repressive regulation of ABA-inducible genes by ZmJAZ14 may shed light on the possible function of JAZ proteins in mediating the JA and ABA interaction.

## Conclusions

In this study, a tassel and endosperm-specific *JAZ* family gene, *ZmJAZ14*, was identified based on microarray data analysis, whose expression was induced by salt and PEG stimuli and exogenous JA, ABA, and GA treatment. ZmJAZ14 may act as a transcriptional regulator since it localized to the nucleus and does not have transcription-activation activity. Transgenic plants overexpressing *ZmJAZ14* showed enhanced tolerance compared to the WT under PEG, JA, and ABA stress conditions, whereas it exhibited a promoted response toward GA stimulus. The expression of many marker genes involved in JA, ABA, and GA signaling pathways were altered in the OE lines. Furthermore, the expression regulation of downstream genes in phytohormone signaling pathways may be attributed to the interaction between ZmJAZ14 and TFs in *Arabidopsis*. These results can be used to further examine the function of JAZ family proteins in maize, and the gene cloned in this study may serve as a candidate for anti-drought and growth promotion regulation in maize.

## Supporting Information

S1 Fig Expression pattern of *JAZ* genes in maize. The expression of *ZmJAZ* genes in different organs and developing seeds was analyzed using microarray. The Affymetrix GeneChip array of maize which contains 17555 probe sets was used. The microarray data were analyzed using a two-class unpaired algorithm method. em, embryo; en, endosperm; se, seed.
(TIF)

S2 Fig Expression levels of Zm*JAZ4* in different maize organs. The relative expression level of *ZmJAZ4* was normalized with *ZmActin1*. The qRT-PCR data were analyzed according to the 2^-ΔΔCt^ method and the error bars indicate standard deviations.
(TIF)

S3 Fig Expression of *ZmJAZ12* and *ZmJAZ20* treated with NaCl, PEG, JA, ABA, GA, and SA in maize seedlings at the three-leaf stage. The maize seedlings were treated with water (control), 250 mM NaCl, 20% PEG, 100 μM JA, 100 μM ABA, 100 μM GA, and 100 μM SA. The shoots and roots were separated and collected. Relative mRNA abundance of *ZmJAZ12* amd *ZmJAZ20* was normalized with the *ZmActin1* gene. The qRT-PCR data were analyzed according to the 2^-ΔΔCt^ method and the error bars indicate standard deviations.
(TIF)

S1 Table The primers used in this study. A word document contains primer sequences used in gene cloning, vector construction and qRT-PCR.
(DOCX)

S2 Table The *cis*-elements in promoter region of *ZmJAZ14*. The putative *cis*-elements in the 2 kb region before the start codon of *ZmJAZ14* were analyzed by Plantcare (http://bioinformatics.psb.ugent.be/webtools/plantcare/html/).
(DOCX)
