# Correction: A Maize Jasmonate Zim-Domain Protein, ZmJAZ14, Associates with the JA, ABA, and GA Signaling Pathways in Transgenic *Arabidopsis*

**DOI:** 10.1371/journal.pone.0128957

**Published:** 2015-06-29

**Authors:** The PLOS ONE Staff

The last author, Lei Wang, should be designated as co-corresponding author with Rumei Chen. Lei Wang’s email address is wanglei01@caas.cn. The publisher apologizes for the error.
