# Supplementary figures and images for: A Maize Jasmonate Zim-Domain Protein, ZmJAZ14, Associates with the JA, ABA, and GA Signaling Pathways in Transgenic Arabidopsis
Source: PLoS One. 2015 Mar 25;10(3):e0121824. doi: 10.1371/journal.pone.0121824 (PMC4373942; doi:10.1371/journal.pone.0121824)

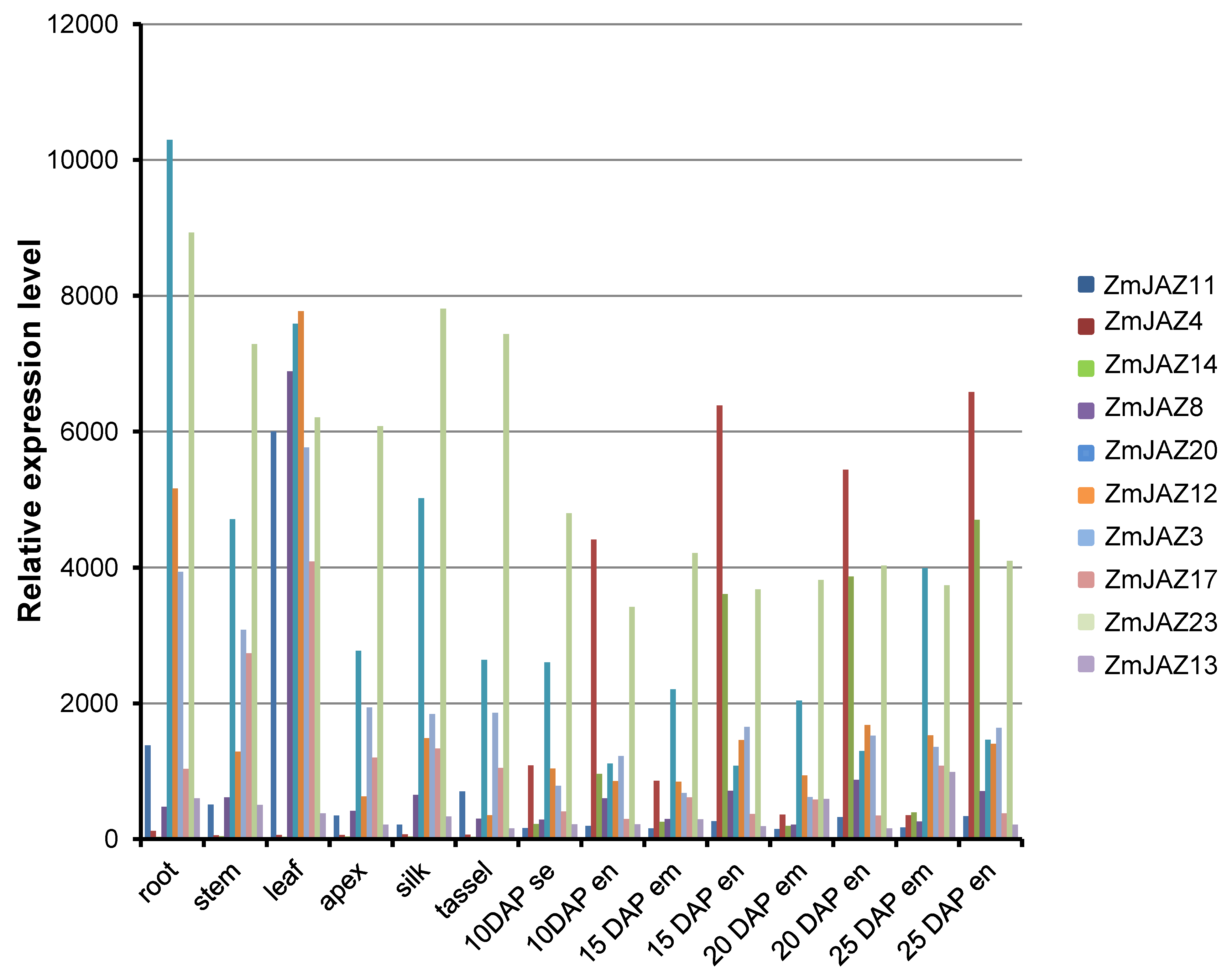

Supplement: S1 Fig — The expression of ZmJAZ genes in different organs and developing seeds was analyzed using microarray. The Affymetrix GeneChip array of maize which contains 17555 probe sets was used. The microarray data were analyzed using a two-class unpaired algorithm method. em, embryo; en, endosperm; se, seed. (TIF) [file pone.0121824.s001.tif]

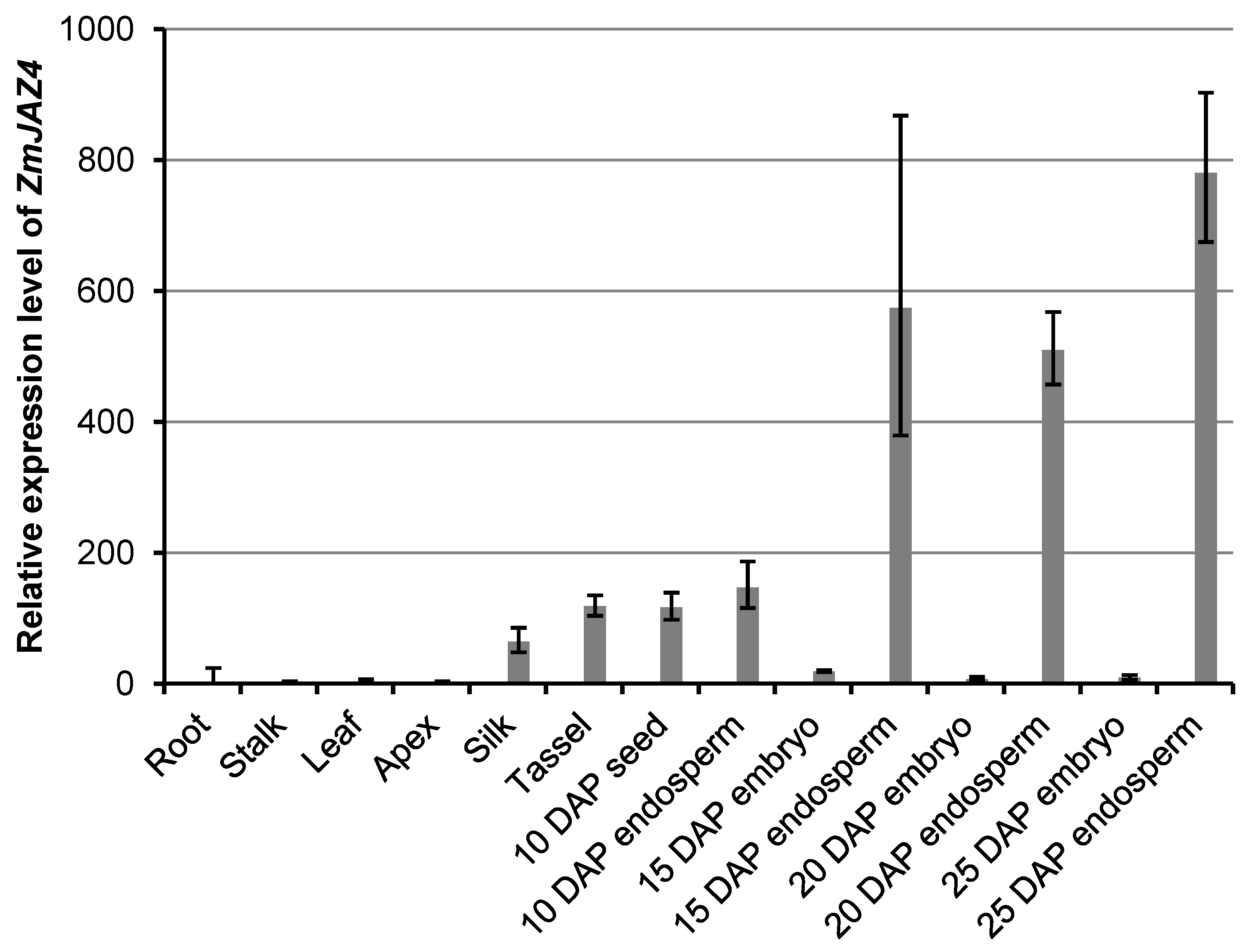

Supplement: S2 Fig — The relative expression level of ZmJAZ4 was normalized with ZmActin1. The qRT-PCR data were analyzed according to the 2-ΔΔCt method and the error bars indicate standard deviations. (TIF) [file pone.0121824.s002.tif]

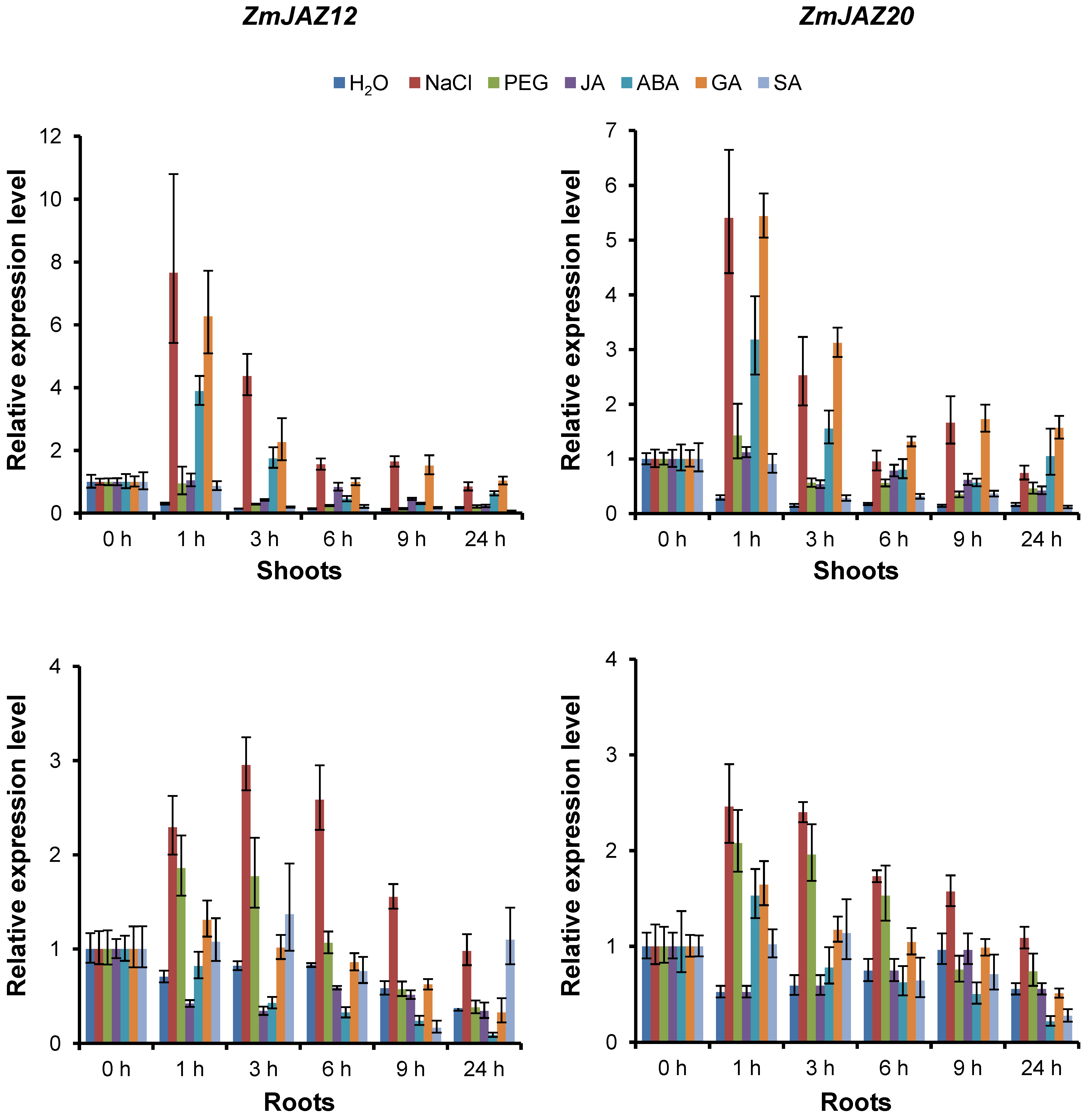

Supplement: S3 Fig — The maize seedlings were treated with water (control), 250 mM NaCl, 20% PEG, 100 μM JA, 100 μM ABA, 100 μM GA, and 100 μM SA. The shoots and roots were separated and collected. Relative mRNA abundance of ZmJAZ12 amd ZmJAZ20 was normalized with the ZmActin1 gene. The qRT-PCR data were analyzed according to the 2-ΔΔCt method and the error bars indicate standard deviations. (TIF) [file pone.0121824.s003.tif]
