# Supplementary material for: A Maize Jasmonate Zim-Domain Protein, ZmJAZ14, Associates with the JA, ABA, and GA Signaling Pathways in Transgenic Arabidopsis
Source: PLoS One. 2015 Mar 25;10(3):e0121824. doi: 10.1371/journal.pone.0121824 (PMC4373942; doi:10.1371/journal.pone.0121824)
Supplement: S1 Table — A word document contains primer sequences used in gene cloning, vector construction and qRT-PCR. (DOCX) [file pone.0121824.s004.docx]

| **Table S1. The primers used in this study** | | |
| --- | --- | --- |
| Genes | Primer Name | Sequence |
| Primers for subcellular localization | | |
| *ZmJAZ14-GFP* | JAZ14GFP-U | 5’- TTCTCGAGATGGCCGCCTCCGGGAC -3' |
|  | JAZ14GFP-L | 5'- TCTAGAGAGCCTGAGCCACGAGGC -3' |
| Primers for transcriptional activation analysis | | |
| *ZmJAZ14-BD* | JAZ14BD-U | 5'- TTGAATTCATGGCCGCCTCCGGGACCGGGAACAAC -3' |
|  | JAZ14BD-L | 5'- TTGGATCCTCAGAGCCTGAGCCACGAGG -3' |
| Primers for the construction of plant expression vectors | | |
| *ZmUBI-*promoter | UBI-U | 5'- TTGATATCTGCAGCGTGACCCGGTCGTGCC -3' |
|  | UBI-L | 5'- TTGGATCCAAGTAACACCAAACAACAGGG -3' |
| *ZmJAZ14-OE* | JAZ14-U | 5'- TTGGATCCATGGCCGCCTCCGGGACCGGGAACAAC -3' |
|  | JAZ14-L | 5'- TTGGTGACCTCAGAGCCTGAGCCACGAGG -3' |
| Primers for qRT-PCR analysis | | |
| *ZmJAZ14* | JAZ14RT-U | 5'- AGGAAGAGGAAGGACAGGATCG -3' |
|  | JAZ14RT-L | 5'- GAAGAGCGGGTTTCAGAGCCT -3' |
| *ZmJAZ4* | JAZ4RT-U | 5'- CCCTTCACCGCTTATTACATGAG -3' |
|  | JAZ4RT-L | 5'- CAGACACGGCATTATTATTATTG -3' |
| *ZmJAZ12* | JAZ12RT-U | 5'- CTGATGCTAAGAAGCCTACTCGC -3' |
|  | JAZ12RT-L | 5'- GCGTCTGAAGGAGAAGTTTGGTA -3' |
| *ZmJAZ20* | JAZ20RT-U | 5'- GAAGAGAAAAGATCGCCTCAATG -3' |
|  | JAZ20RT-L | 5'- GGTTCAGGCTGGAATCCACGGCA -3' |
| *ZmActin1* | ZmActin1-U | 5'- ATGTTTCCTGGGATTGCCGAT -3' |
|  | ZmActin1-L | 5'- CCAGTTTCGTCATACTCTCCCTTG -3' |
| *AtPDF1.2* | AtPDF1.2-U | 5’- GTTCTCTTTGCTGCTTTCGAC -3’ |
|  | AtPDF1.2-l | 5’- GCAAACCCCTGACCATGT -3’ |
| *AtVSP2* | AtVSP2-U | 5’- ACGACTCCAAAACCGTGTGCAA -3’ |
|  | AtVSP2-L | 5’- CGGGTCGGTCTTCTCTGTTCCGT -3’ |
| *AtNADP-ME* | AtNADP-ME-U | 5’- TGGTCTGATCTACCCGCCATT -3’ |
|  | AtNADP-ME-L | 5’- CGCCAATCCGAGGTCATAGG -3’ |
| *AtEM1* | AtEM1-U | 5’- TCAAATGGTATGCGGTTATG -3’ |
|  | AtEM1-L | 5’- TATCACAAGTAAGACACGAAG -3’ |
| *AtEM6* | AtEM6-U | 5’- TGTCTCGTTTGTTTTCCAG -3’ |
|  | AtEM6-L | 5’- CACTATGTTGAGAATCCAC -3’ |
| *AtGA20ox1* | AtGA20ox1-U | 5’- GGCATCAGCGAGGAGCTTATT -3’ |
|  | AtGA20ox1-L | 5’- CTGCTTGCGTAGCCAACACT -3’ |
| *AtActin2* | AtActin-U | 5’- TAACTCTCCCGCTATGTATGTCGC -3’ |
|  | AtActin-L | 5’- GAGAGAAACCCTCGTAGATTGGC -3’ |
| Primers for yeast two-hybrid assays | | |
| *AtMYC2* | AtMYC2-U | 5’- CATATGATGACTGATTACCGGCTACAACCA -3’ |
|  | AtMYC2-L | 5’- AGATCTACCGATTTTTGAAATCAAACTTGC -3 |
| *AtMYC3* | AtMYC3-U | 5’- CATATGATGAACGGCACAACATCATCAA -3’ |
|  | AtMYC3-L | 5’- GGATCCATAGTTTTCTCCGACTTTCGTCATC -3’ |
| *AtGL3* | AtGL3-U | 5’- GAATTCATGGCTACCGGACAAAACAGAA -3’ |
|  | AtGL3-L | 5’- GGATCCACAGATCCATGCAACCCTTTGA -3’ |
| *AtEGL3* | AtEGL3-U | 5’- GAATTCATGGCAACCGGAGAAAACAGA -3’ |
|  | AtEGL3-L | 5’- GGATCCACATATCCATGCAACCCTTTGAA -3’ |
| *AtPAP1* | AtPAP1-U | 5’- GAATTCATGGAGGGTTCGTCCAAAGG -3’ |
|  | AtPAP1-L | 5’- GGATCCATCAAATTTCACAGTCTCTCCATCG -3’ |
| *AtGL1* | AtGL1-U | 5’- GAATTCATGAGAATAAGGAGAAGAGATGAAAAAG -3’ |
|  | AtGL1-L | 5’- GGATCCAAGGCAGTACTCAACATCACCAGA -3’ |
| *AtMYB21* | AtMYB21-U | 5’- CATATGATGGAGAAAAGAGGAGGAGGAAGT -3’ |
|  | AtMYB21-L | 5’- GGATCCATTACCATTCAATAAATGCATTGATG -3’ |
| *AtMYB24* | AtMYB24-U | 5’- GAATTCATGGAGAAAAGAGAAAGTAGTGGTG -3’ |
|  | AtMYB24-L | 5’- GGATCCATTACCATTATATATATTCATGGGCC -3’ |
| *AtEIN3* | AtEIN3-U | 5’- ATCGATATGATGTTTAATGAGATGGGAATGT -3’ |
|  | AtEIN3-L | 5’- GGATCCGAACCATATGGATACATCTTGCTG -3’ |
| *AtEIL1* | AtEIL1-U | 5’- GAATTCATGATGATGTTTAACGAGATGGGA -3’ |
|  | AtEIL1-L | 5’- GGATCCGAACCATATTGATACATCTTGCTGC -3’ |
| *AtGAI* | AtGAI-U | 5’- GAATTCATGAAGAGAGATCATCATCATCATCA -3’ |
|  | AtGAI-L | 5’- GGATCCATTGGTGGAGAGTTTCCAAGC -3’ |
| *AtRGA* | AtRGA-U | 5’- CCCGGGATGAAGAGAGATCATCACCAATTCC -3’ |
|  | AtRGA-L | 5’- GGATCCGTACGCCGCCGTCGAGAGTTT -3’ |
| *AtRGL1* | AtRGL1-U | 5’- CATATGATGAAGAGAGAGCACAACCACCG -3’ |
|  | AtRGL1-L | 5’- GGATCCTTCCACACGATTGATTCGCCA -3’ |
| *AtRGL3* | AtRGL3-U | 5’- CATATGATGAAACGAAGCCATCAAGAAA -3’ |
|  | AtRGL3-L | 5’- AGATCTCCGCCGCAACTCCGCCGCTA -3’ |
| *AtNINJA* | AtNINJA-U | 5’- GAATTCATGGACGATGATAATGGGCTCG -3’ |
|  | AtNINJA-L | 5’- AGATCTGGTGTGAGCTGACGCTGCAGT -3’ |
| *AtHDA6* | AtHDA6-U | 5’- GAATTCATGGAGGCAGACGAAAGCGG -3’ |
|  | AtHDA6-L | 5’- CTCGAGAGACGATGGAGGATTCACGTCTG -3’ |
| *AtJAZ1* | AtJAZ1-U | 5’- GAATTCATGTCGAGTTCTATGGAATGTTCTG -3’ |
|  | AtJAZ1-L | 5’- GGATCCTATTTCAGCTGCTAAACCGAGC -3’ |
| *AtJAZ2* | AtJAZ2-U | 5’- GAATTCATGTCGAGTTTTTCTGCCGAGT -3’ |
|  | AtJAZ2-L | 5’- GGATCCCCGTGAACTGAGCCAAGCTG -3’ |
| *AtJAZ3* | AtJAZ3-U | 5’- GAATTCATGGAGAGAGATTTTCTCGGGTT -3’ |
|  | AtJAZ3-L | 5’- CTCGAGGGTTGCAGAGCTGAGAGAAGAAC -3’ |
| *AtJAZ4* | AtJAZ4-U | 5’- GAATTCATGGAGAGAGATTTTCTCGGGC -3’ |
|  | AtJAZ4-L | 5’- CTCGAGGTGCAGATGATGAGCTGGAGG -3’ |
| *AtJAZ5* | AtJAZ5-U | 5’- GAATTCATGTCGTCGAGCAATGAAAATG -3’ |
|  | AtJAZ5-L | 5’- GGATCCTAGCCTTAGATCGAGATCTTTCGA -3’ |
| *AtJAZ6* | AtJAZ6-U | 5’- GAATTCATGTCAACGGGACAAGCGC -3’ |
|  | AtJAZ6-L | 5’- GGATCCAAGCTTGAGTTCAAGGTTTTTGGA -3’ |
| *AtJAZ7* | AtJAZ7-U | 5’- GAATTCATGATCATCATCATCAAAAACTGCGA -3’ |
|  | AtJAZ7-L | 5’- CTCGAGTCGGTAACGGTGGTAAGGGGA -3’ |
| *AtJAZ8* | AtJAZ8-U | 5’- CATATGATGAAGCTACAGCAAAATTGTGACT -3’ |
|  | AtJAZ8-L | 5’- AGATCTTCGTCGTGAATGGTACGGTGA -3’ |
| *AtJAZ9* | AtJAZ9-U | 5’- GAATTCATGGAAAGAGATTTTCTGGGTTT -3’ |
|  | AtJAZ9-L | 5’- GGATCCTGTAGGAGAAGTAGAAGAGTAATTCATT -3’ |
| *AtJAZ10* | AtJAZ10-U | 5’- GAATTCATGTCGAAAGCTACCATAGAACTC -3’ |
|  | AtJAZ10-L | 5’- GGATCCGGCCGATGTCGGATAGTAAG -3’ |
| *AtJAZ11* | AtJAZ11-U | 5’- GAATTCATGGCTGAGGTAAACGGAGATT -3’ |
|  | AtJAZ11-L | 5’- GGATCCTGTCACAATGGGGCTGGTTTC -3’ |
| *AtJAZ12* | AtJAZ12-U | 5’- CATATGATGACTAAGGTGAAAGATGAGCCA -3’ |
|  | AtJAZ12-L | 5’- GGATCCAGCAGTTGGAAATTCCTCCTTG -3’ |
| Primers for bimolecular fluorescence complementation analysis | | |
| *ZmJAZ14* | JAZ14-BiFC-U | 5’- TCTAGAATGGCCGCCTCCGGGAC -3' |
|  | JAZ14-BiFC-L | 5’- CTCGAGCCTGAGCCACGAGGC -3' |
| *AtMYC2* | AtMYC2-BiFC-U | 5’- ACTAGTATGACTGATTACCGGCTACAACCA -3' |
|  | AtMYC2-BiFC-U | 5’- GTCGACACCGATTTTTGAAATCAAACTTGC -3' |
| *AtMYC3* | AtMYC3-BiFC-U | 5’- ACTAGTATGTCTCCGACGAATGTTCAAGT -3' |
|  | AtMYC3-BiFC-L | 5’- CTCGAGTGGACATTCTCCAACTTTCTCCGT -3' |
| *AtMYB21* | AtMYB21-BiFC-U | 5’- TCTAGAATGGAGAAAAGAGGAGGAGGAAGT -3' |
|  | AtMYB21-BiFC-L | 5’- CTCGAGATTACCATTCAATAAATGCATTGATGACCA -3' |
| *AtNINJA* | AtNINJA-BiFC-U | 5’- TCTAGAATGGACGATGATAATGGGCTCGAGCTCAGC -3' |
|  | AtNINJA-BiFC-L | 5’- GTCGACGGTGTGAGCTGACGCTGCAGTCATT -3' |
