# Supplementary material for: A Maize Jasmonate Zim-Domain Protein, ZmJAZ14, Associates with the JA, ABA, and GA Signaling Pathways in Transgenic Arabidopsis
Source: PLoS One. 2015 Mar 25;10(3):e0121824. doi: 10.1371/journal.pone.0121824 (PMC4373942; doi:10.1371/journal.pone.0121824)
Supplement: S2 Table — The putative cis-elements in the 2 kb region before the start codon of ZmJAZ14 were analyzed by Plantcare (http://bioinformatics.psb.ugent.be/webtools/plantcare/html/). (DOCX) [file pone.0121824.s005.docx]

**Table S2. The *cis*-elements in promoter region of *ZmJAZ14***

| Site name | Position | Strand | Sequence | Function |
| --- | --- | --- | --- | --- |
| ABRE  CAT-box  CGTCA-motif  MBS  P-box  RY-element  Skn-1_motif  TC-rich repeats  TGACG-motif | 1311  1242  211  559  1191  711  235, 759  696  211 | +  -  +  -  -  +  +, -  -  - | CACGTG  GCCACT  CGTCA  CAACTG  CCTTTTG  CATGCATG  GTCAT  ATTTTCTTCA  TGACG | abscisic acid responsiveness  meristem expression  MeJA-responsiveness  drought-inducibility  gibberellin-responsive element  seed-specific regulation  endosperm expression  defense and stress responsiveness  MeJA-responsiveness |

An *in silico* promoter analysis of *ZmJAZ14* were made with Plantcare (<http://bioinformatics.psb.ugent.be/webtools/plantcare/html/>).
